# NK homeobox 2.2 functions as tumor suppressor in colorectal cancer due to DNA methylation

**DOI:** 10.7150/jca.43665

**Published:** 2020-06-06

**Authors:** Yuan He, Xiao-Yun Liu, Rui Gong, Kun-Wei Peng, Rong-Bin Liu, Fang Wang

**Affiliations:** 1 State Key Laboratory of Oncology in South China, Collaborative Innovation Center for Cancer Medicine, Sun Yat-sen University Cancer Center, Guangzhou 510060, China.; 2 Department of Ultrasound and Electrocardiogram, Sun Yat-sen University Cancer Center, Guangzhou 510060, China.; 3 Department of Molecular Diagnostics, Sun Yat-sen University Cancer Center, Guangzhou 510060, China.; 4 Department of Healthcare, Union Hospital, Tongji Medical College, Huazhong University of Science and Technology, Wuhan 430022, China.; 5 Department of Ultrasound, Sun Yat-sen Memorial Hospital, Sun Yat-sen University, Guangzhou 510120, China.

**Keywords:** Colorectal cancer, Hypermethylation, NK homeobox 2.2, Epigenetic

## Abstract

**Aim:** The role of NK homeobox 2.2 (NKX2.2) in human colorectal cancer (CRC) remains to be unveiled. This study was designed to explore the epigenetic regulation and function of NKX2.2 in human CRC.

**Methods:** The Cancer Genome Atlas (TCGA) and Gene Expression Omnibus (GEO) datasets were used to assess the methylation data of NKX2.2 in CRC. Six CRC cell lines (HCT116, SW480, HT29, LOVO, SW1116, SW640) and 20 pairs of primary CRC tumor and normal tissues were utilized to explore the function of NKX2.2 in CRC using Sequenom EpiTYPER®, verified by cloning-based bisulfite sequencing analysis, semi-quantitative reverse transcription PCR, western blot, cell viability assessment, plate clone formation assay , and transwell assays.

**Results:** Bioinformatic analysis showed that NKX2.2 was significantly hypermethylated in primary tumors compared to normal tissues (p < 0.05). Our study also found that NKX2.2 methylation was upregulated (p<0.05) in tumors than normal tissues. In vitro experiments demonstrated that 5-aza-2'-deoxycytidine downregulated the methylation of NKX2.2 and retrieved its expression of mRNA and protein levels (p<0.05). No significant association was found between the NKX2.2 methylation and sex, age, tumor differentiation, TNM stage, CEA, CA199, and fecal occult blood (p>0.05). Kaplan-Meier analysis indicated that NKX2.2 hypermethylation showed a trend but not statistical significance for predicting poor overall survival in CRC patients (p=0.33). NKX2.2 overexpression suppressed cell proliferation, colony formation, and inhibited tumor invasion and migration in CRC cells (both p<0.05).

**Conclusions:** This study indicates that NKX2.2 is a tumor suppressor in CRC due to hypermethylation.

## Introduction

Though developments in colorectal cancer (CRC) diagnosis and therapy have been made in the past few decades, much work is required as it remains one of the leading causes of cancer-related mortality with high incidence worldwide 1. DNA methylation is a common epigenetic modification that has heritable variations in gene expression not encoded by the DNA sequence. A large number of studies have been reported to characterize methylation modification during the process of normal to CRC 2. Nowadays, the methylation therapy is promising and deserved to explore furtherly in the CRC development as well as progression.

The NK homeobox 2 family transcription factors include Nkx2.1, Nkx2.2, Nkx2.3, Nkx2.4, Nkx2.5, Nkx2.6 and Nkx2.8, which bind to DNA on unique consensus sequence T(C/T) AAGTG 3. The NK homeobox 2.2 (NKX2.2), located in chormosome chromosome number 20, acts as transcription factor with tissue-specific distributions 4. It plays a crucial role in the development of central nervous system and differentiation of oligodendrocyte and neuroendocrine in the gastrointestinal tract and pancreas 5-8. NKX2.2 functions as a transcription repressor by recruitment of co-repressor Groucho 3 or Groucho 4, but it also contains a transcriptional activation domain, which is regulated by the conserved Nk2-specfic domain. Moreover, NKX2.2 contains a homeodomain, which is responsible for its DNA binding activity and the subcellular distribution of this protein 9. Abnormalities in NKX2.2 gene has been associated with various cancers, including brain tumor 10, Ewing sarcoma 11, Hodgkin lymphoma 12, neuroendocrine tumors 13, small cell lung cancer^14^, and osteosarcoma 15. Besides, NKX2.2 is reported to be methylated in cervical cancer and luminal breast cancers 16, 17. However, the function role of NKX2.2 in CRC remains unknown. Here we aim to investigate the role and clinical significance of NKX2.2 methylation in CRC.

## Materials and Methods

### Bioinformative analysis of database

#### NKX2.2 methylation data

The methylation data was obtained and analyzed from TCGA using Infinium HumanMethylation450 BeadChip® microarrays (Illumina Inc., San Diego, CA, USA). The genomic coordinates of the CpGs are based on GRCh37. All methylation values are expressed as β values (β = the methylated probe intensity / the overall intensity). Differential methylation analysis of level-3 CRC data from TCGA was carried out using SMART with default parameters. Subsequently, 274 differentially methylated CpGs were found. Then the absolute β-difference values were calculated by subtracting normal β-values from tumor β-values for each sample pairs. As a result, NKX2.2 gene was selected as a candidate for further study.

#### NKX2.2 validation datasets

To further validate the diagnostic role of NKX2.2 methylation in CRC, logistic regression model training of TCGA and validation of GEO data was performed with R statistics using the pROC package. Three additional methylation datasets were downloaded from the GEO (GSE42752, GSE77718, and GSE101764).

#### NKX2.2 mRNA expression data

TCGA expression data (level 3) were obtained and analyzed using the IlluminaHiSeq_RNASeqV2 platform (Illumina, San Diego, CA, USA). The NKX2.2 transcript ENSG00000125820.5 was the most abundant in the ribonucleic acid sequencing (RNA-seq) data. RNA-Seq by fragments per kilobase of exon model per million reads mapped (FPKM) was used as the algorithm for quantifying transcript abundances from RNA-seq data. All RNA-seq expression values are log^2^ transformed.

### CRC clinical samples and cell lines

Twenty pairs of fresh frozen CRC tissues (tumor and corresponding adjacent) were obtained from the Sun Yat-sen University Cancer Center (SYSUCC), Guangzhou, China, between December 2015 and June 2017. All samples were selected and confirmed histologically based on the following criteria: primary resection of the tumor with detailed follow-up data and no preoperative anticancer treatment. The human CRC cell lines (HCT116, SW480, HT29, LOVO, SW1116, SW640) and controlled cell line (HEK-293T) were obtained from the Chinese Academy of Sciences, Shanghai Institutes for Cell Resource Center. HCT116 and HT29 were maintained in McCoy's 5A with L-glutamine (HyClone, Logan, UT, USA). SW480 was cultured in incomplete Leibovitz's L-15 medium (KeyGEN, Nanjing, Jiangsu, China) media supplemented with 10% heat-inactivated fetal bovine serum (Gibco, Carlsbad, CA, USA). The LOVO, SW1116, SW640, and HEK-293T were supported with DMEM (Gibco, Carlsbad, CA, USA) with 10% heat-inactivated fetal bovine serum (Gibco, Carlsbad, CA, USA). Cells were grown in a humidified incubator at 37 °C with 5% CO_2_. This present study was approved by the Ethics Committee of Sun Yat-sen University Cancer Center (NO. L102012020000B). All participants were recruited after providing signed informed consent.

### DNA isolation and sequenom EpiTYPER^®^ analysis

Genomic DNA was extracted from the tissue samples and cell lines using a nucleic acid isolation kit (Qiagen, Hilden, NW, Germany). Then the DNA was subjected to bisulfite treatment using the EZ conversion kit (Zymo Research, Orange, CA, USA) according to the manufacturer's instructions. Methylation of NKX2.2 was quantitatively analyzed using the Sequenom MassArray platform (Agena Bioscience, San Diego, CA, USA), which employs matrix-assisted laser-desorption/ionization time-of-flight mass spectrometry and RNA base-specific cleavage. The NKX2.2 sense primer 5'-GGATGAGGTTGGTTAGGTGT-3' and anti-sense primer 5'-CCCTCAAAACTCAAACTCCAAAT-3', were used. Each forward and reverse primer was modified with a 10-mer tag (5′-AGGAAGAGAG-3′) or a T7-promoter tag (5'-CAGTAATACGACTCACTATAGGGAGAAGGCT-3′) for transcription in vitro. A total of 24 CpG sites in this region were checked. The spectra methylation ratios were generated on EpiTYPER 1.0 (Agena Bioscience, San Diego, CA, USA).

### Bisulfite-specific PCR (BSP) and Sequencing

The bisulfite-treated DNA was amplified by PCR for NKX2.2 gene with bisulfite special PCR (BSP) specific primer pair (Forward: 5'-GGATGAGGTTGGTTAGGTGT-3'; Reverse: 5'-CCCTCAAAACTCAAACTCCAAAT-3') under the following conditions: 94 °C denaturation for 3 min, 40 cycles of 94 °C for 30 s, 52 °C for 30 s, 72 °C for 30 s, and 72 °C elongation for 5 min.The PCR products were separated by electrophoresis in 1.5% agarose gel with ethidium bromide. Bands were isolated from the gel and purified with Wizard SV Gel and PCR Clean-Up System (Tiangen, Beijing, China). Following this, the purified PCR products were subcloned into a TOPO Vector (Ruibo, Guangzhou, Guangdong, China) according to the manufacturer's instructions. Positive clones were obtained by ampicillin selection followed by PCR colony screening. Six positive clones were randomly selected for sequencing at Ruibo (Ruibo, Guangzhou, Guangdong, China).

### Demethylation by 5-Aza-2'-deoxycytidine (5-AZC) treatment

For validation of the role of epigenetics in transcriptional regulation, HCT116, and SW480 were used to conduct an in vitro functional study of NKX2.2 on 6-well plates. Briefly, 1 × 10^5^ cells were incubated with demethylating agent 5-AZC (0 μmol/L vs 5 μmol/L vs 10 μmol/L) and with media changed every 24 hours for 72 hours.

### RNA extraction and qRT-PCR

Trizol (Invitrogen, Carlsbad, CA, USA) was used to purify the total RNA of cell lines. Reverse transcription and qRT-PCR were performed using a SYBR Green qRT-PCR kit (Takara, Shiga, Japan) and CFX96 real-time polymerase chain reaction (PCR) detection system (Bio-Rad, Hercules, CA, USA) in triplicate. GAPDH was used as an internal reference. The primers used were synthesized by Ruibo (Ruibo, Guangzhou, Guangdong, China) and were as follows: NKX2.2 (Forward: 5'- CTCGAACCATGTCGCTGACCAA-3' and Reverse: 5'- CCACAGAGCCCTCCTCATCGT -3') and GAPDH (Forward: 5'-GCACCGTCAAGGCTGAGAAC-3' and Reverse: 5'- TGGTGAAGACGCCAGTGGA -3'). Experiments were repeated in triplicate.

### Protein preparation and western blot

The cells were lysed in ice-cold RIPA and a protease inhibitor cocktail (Beyotime, Shanghai, China). Protein concentrations were quantified using the BCA protein assay kit (Beyotime, Shanghai, China). The protein lysates were then separated by dodecyl sulfate sodium salt - polyacrylamide gel electrophoresis and electroblotted onto polyvinylidene fluoride membranes (Merck, NJ, USA). After blocking with 5% nonfat milk in Tris-buffered saline, the membranes were incubated with antibodies (NKX2.2 (1:250, Proteintech, China), and β-actin (1:1000, Santa cruz biotechnology, USA). Rabbit anti-β-actin antibody was used as a reference control. The results were visualized using the Bio-rad chemiDocTouch chemiluminescence imaging system instrument (Bio-rad, USA).

### Plasmid construction and transfection

The human full-length NKX2.2 cDNA (NM_002509) was cloned into the pEZ-M35 vector. The Sequencing primers were as follows: 5′-AGGCACTGGGCAGGTAAG-3′ (forward) and 5′-GTGGCACCTTCCAGGGTC-3′ (reverse). The NKX2.2 overexpression plasmid (pEZ-M35-NKX2.2) and the negative control plasmid (pEZ-M35-Vector) were synthesized by Guangzhou GeneCopoeia Co., Ltd, respectively. Lipofectamine 3000 (Invitrogen, USA) was used for transfection according to the manufacturer's instructions, which was lasted for 48 h. After transfection, the cells were collected for further analysis.

### Cell viability assessment

For cell viability, 1*10^3^ cells / well were seeded in 96-well plates and the cell viability was measured by the Cell Counting Kit-8 (Dojindo, Kumamoto, Japan) at 0 h vs 12 h vs 24 h vs 48 h vs 72 h. Experiments were repeated at least three times. Absorbance was measured on a microplate reader (Tecan, Switzerland) at a wavelength of 490 nm.

### Migration and invasion assays

For cell migration and invasion, cells (2*10^5^) were placed into the upper chamber of a transwell apparatus coated with extracellular matrix gel (ECM gel, BD Biosciences, San Jose, CA) and incubated for 24 hours. Cells that invaded into the lower membrane surface were stained with crystal violet (Sangon, Shanghai, China) and counted in three independent fields by Image J.

### Plate clone formation assay

Transfected cells were collected and inoculated into a 6-well plate at a concentration of 500 cells / dish. The cell clones were cultured for 2 weeks until visible cell clones emerged. Fresh medium was replaced every 3 days. The cells were gently washed with PBS twice and fixed with 4% paraformaldehyde (Biosharp, Hefei, China) for 20 min at room temperature, and stained with crystal violet (Sangon, Shanghai, China) for 20 min at room temperature. Each cell clone on the dishes was counted and photographed triplicately.

### Statistical analysis

We used R software and SPSS 20.0 (IBM Corporation, Armonk, NY) to perform statistical analyses. Student's t-test, an Anova test, and *χ*^2^ test were utilized to analyze the categorical or continuous variables, respectively. Moreover, Spearman's correlation was used to compare the linear association between the methylation and transcription level. ROC curve was also plotted for the diagnostic comparison of CRC. The kaplan-meier analysis was used to assess the potential independent prognostic factors. All tests were 2-sided at 0.05.

## Results

### Bioinformative evaluation of NKX2.2 methylation and mRNA expression

**Figure 1:**
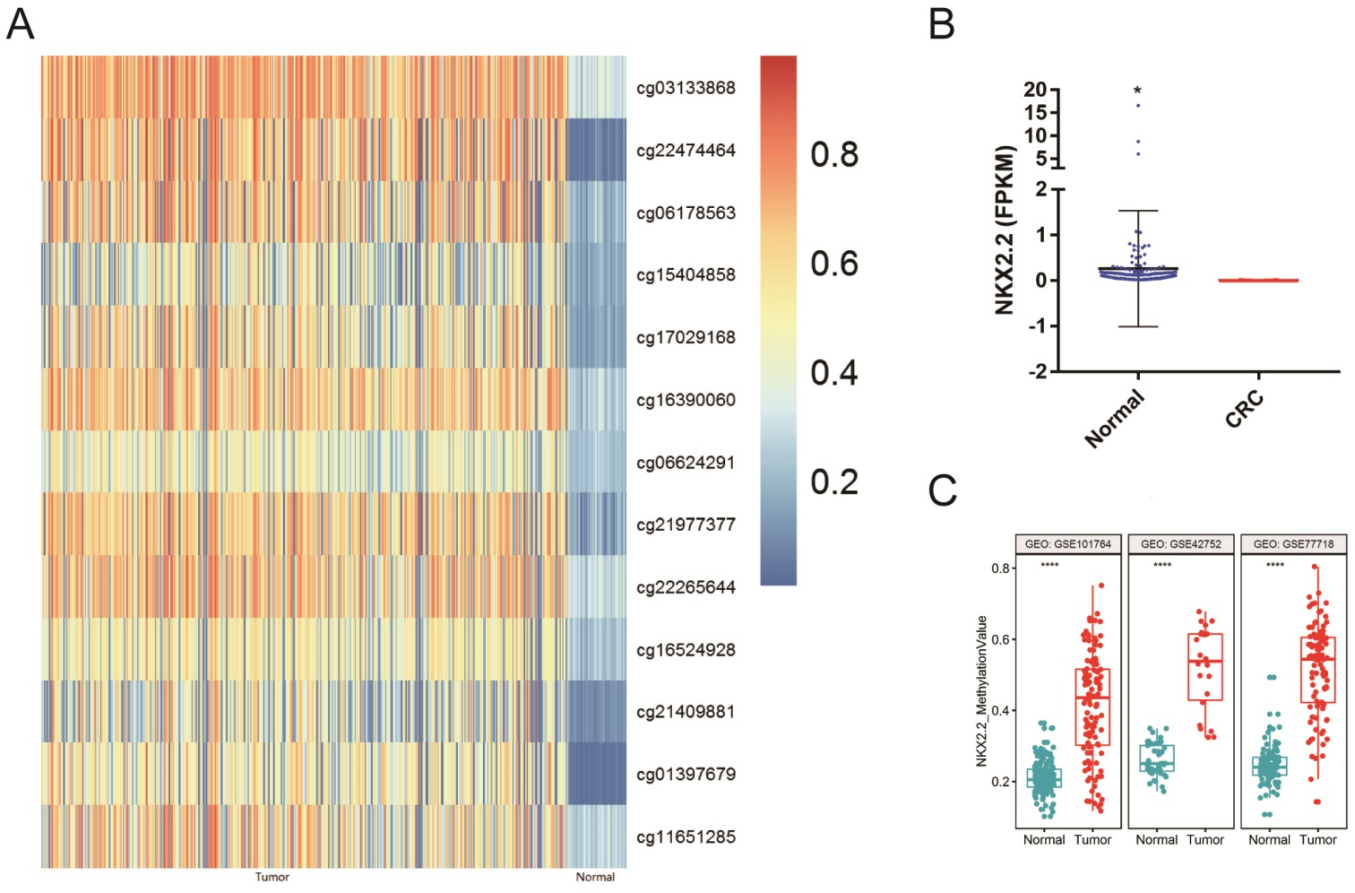
Bioinformative evaluation of NKX2.2 methylation and mRNA Expression in TCGA and GEO databases: **(A)** The methylation level of NKX2.2 was significantly upregulated in colorectal cancer compared to normal tissues (p<0.05); **(B)** The mRNA expression of NKX2.2 was significantly downregulated in colorectal cancer than normal tissues (fold change ≥2, *p<0.05) ; **(C)** Three additional GEO methylation datasets demonstrated that the levels of NKX2.2 methylation were significantly higher in colorectal cancer samples compared to normal colorectal mucosa samples (****p < 0.0001).

The methylation and mRNA expression of NKX2.2 in CRC were explored in TCGA database. As depicted in Figure 1A, the methylation level of NKX2.2 was found to be significantly upregulated in CRC compared to normal tissues (p < 0.05). Meanwhile, the mRNA expression of NKX2.2 was found to be significantly downregulated in CRC (fold change ≥2, p < 0.05, Figure 1B).

To further validate the methylation status of NKX2.2 in human CRC, three additional methylation datasets were downloaded from the GEO datasets. The results also demonstrated that the levels of NKX2.2 methylation were significantly higher in CRC samples compared to normal colorectal mucosa samples (p < 0.0001, Figure 1C).

### NKX2.2 is hypermethylated in human primary CRC compared to normal tissues

**Figure 2:**
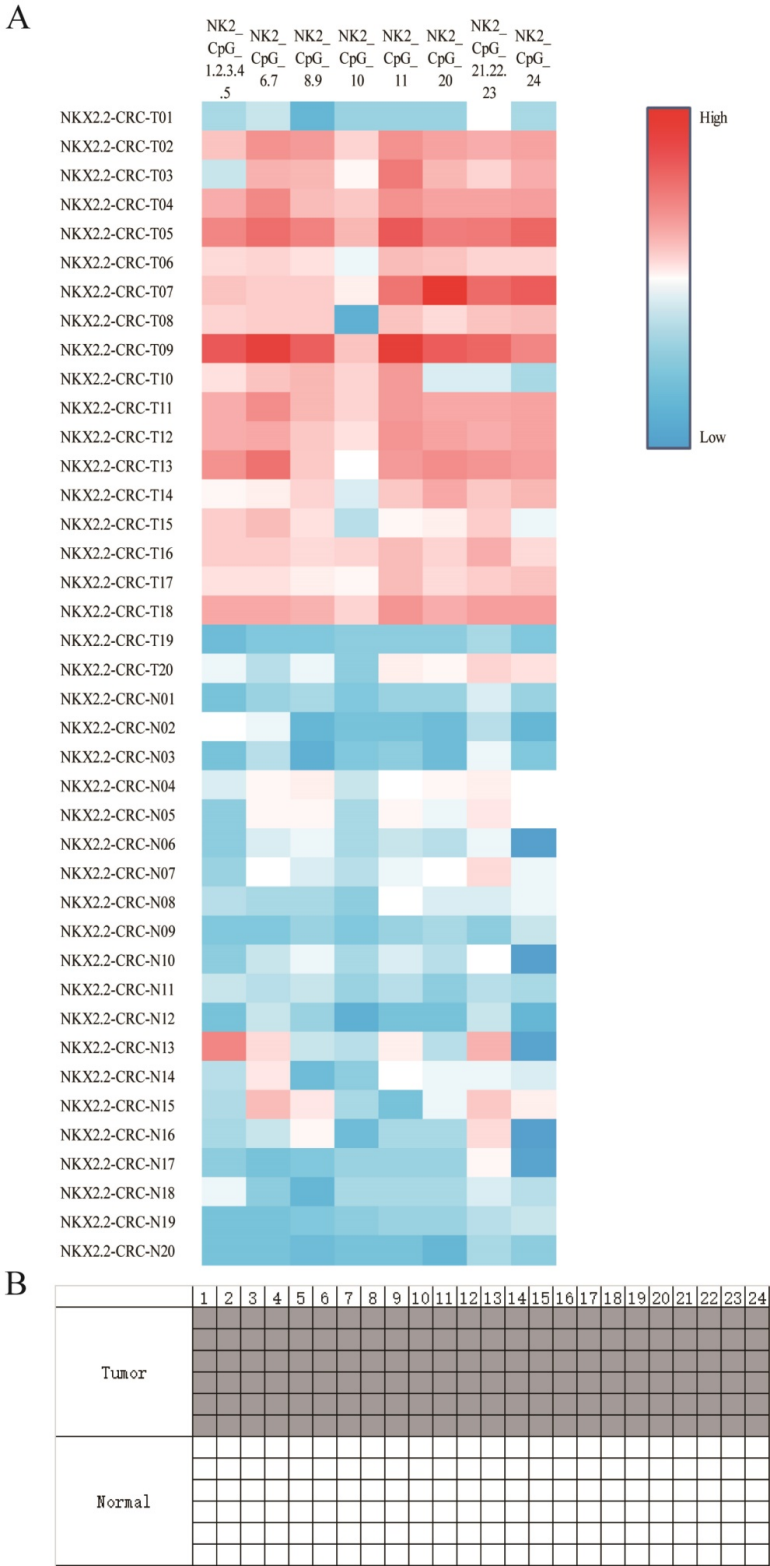
**(A).** Sequenom EpiTYPER system analysis of the average methylation ratio of NKX2.2 in colorectal cancer and normal tissues, p<0.05. **(B)** Representative NKX2.2 methylation levels between tumor and normal sample using bisulfite special PCR based sequencing analysis. The tumor cell sample was higher and the control sample was lower. Each line individually sequenced clone, and each square CpG residue. White and black squares were unmethylated and methylated cytosines, respectively (p<0.05).

To investigate the biological function of NKX2.2 methylation, 20 pairs of CRC tissues and corresponding adjacent normal tissues were included from Sun Yat-sen University Cancer Center. The clinicopathological features were summarized in Table 1. NKX2.2 was significantly hypermethylated in tumor compared to normal tissues (0.24±0.09 vs 0.11±0.03, p < 0.001, Figure 2A), subgroup analysis showed statistical differences in each CpG site (all p < 0.05). Meanwhile, representative results of BSP were consistent with the results described above (Figure 2B). Moreover, there was no significant correlation between NKX2.2 methylation and clinicopathological parameters, such as sex, age, tumor differentiation, TNM stage, CEA, CA199, and fecal occult blood (p > 0.05, Table 1).

### Receiver operating characteristic (ROC) analysis of NKX2.2 methylation in CRC

**Figure 3:**
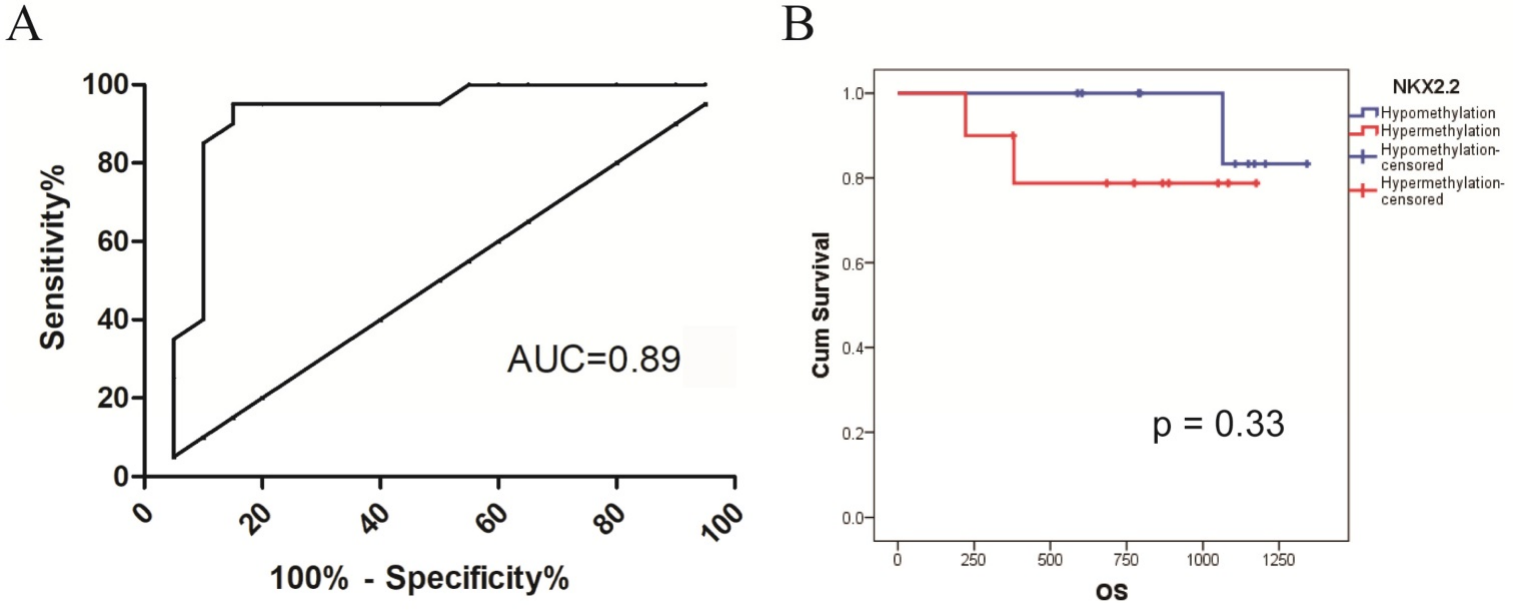
** (A)** Receiver operating characteristic analysis of NKX2.2 methylation in CRC: the AUC, sensitivity and specificity of NKX2.2 methylation were 0.89, 90.0%, 85.0%, respectively. **(B)** Kaplan-Meier survival analysis of NKX2.2 methylation for overall survival in colorectal cancer (p = 0.33).

ROC curve analysis was utilized to test the sensitivity and specificity of NKX2.2 methylation for the diagnostic performance of CRC. The best cut-off value of average NKX2.2 methylation ratio was 0.15 according to Youden index. The area under the curve (AUC), sensitivity and specificity of NKX2.2 methylation were 0.89, 90.0%, 85.0%, respectively (Figure 3A).

### Identification of NKX2.2 methylation associated with prognosis in CRC

To investigate its prognostic value in CRC, we divided the patients into two groups. Follow-up data was available in 20 patients with a survival time ranged from 222 days to 1341 days (median 878 days). By Kaplan-Meier analysis, NKX2.2 hypermethylation showed a trend but not statistical significance for predicting poor overall survival in CRC patients (p = 0.33, Figure 3B).

### Regulation of NKX2.2 expression by DNA methylation in human CRC cell lines

**Figure 4:**
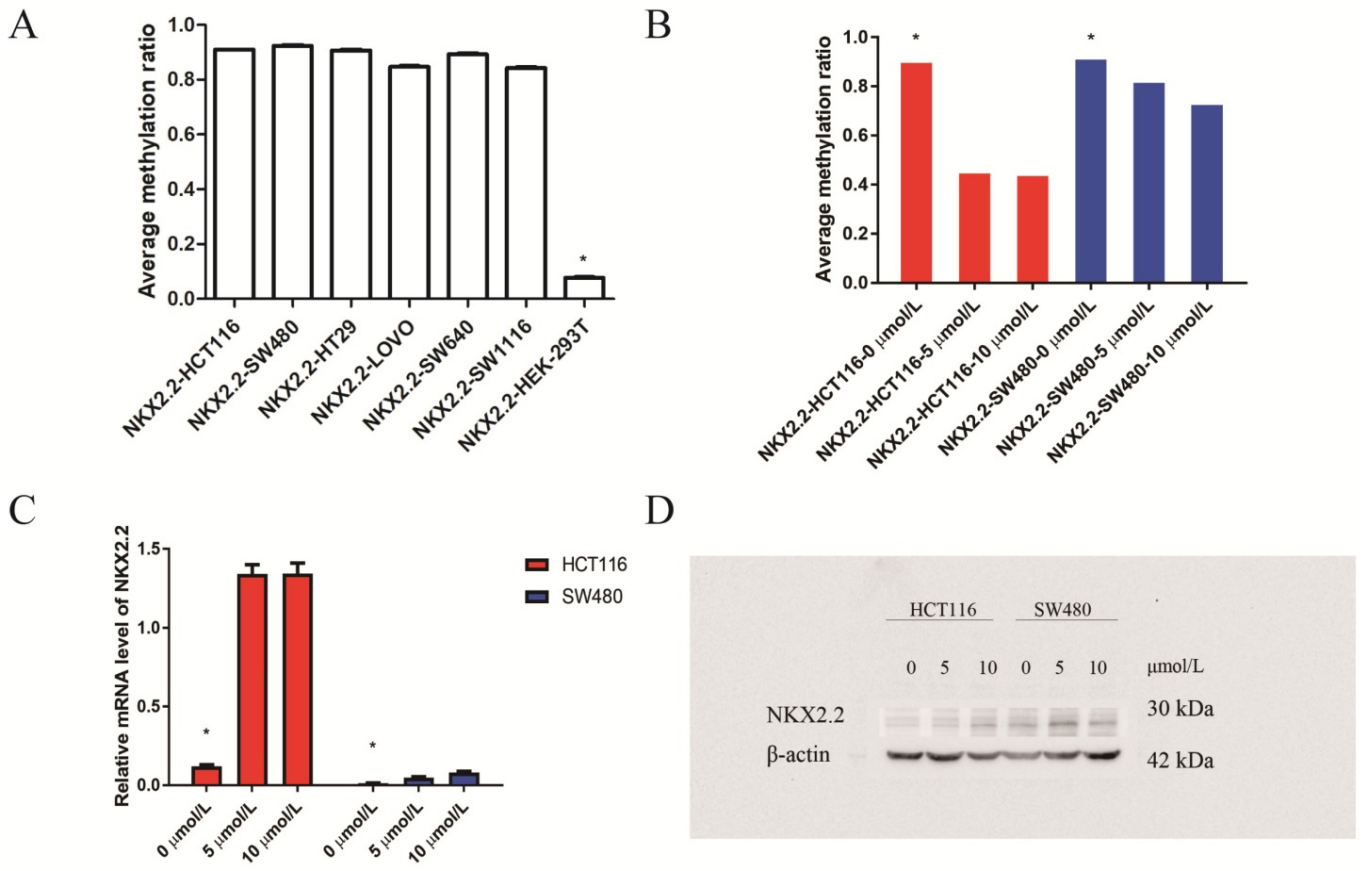
** (A)** Sequenom EpiTYPER system analysis of the average methylation ratio of NKX2.2 in colorectal cancer (CRC) cell lines(HCT116, SW480, SW640, HT29, SW1116, LOVO ) and reference cell line (HEK-293T) (p<0.05); **(B)** Sequenom EpiTYPER system analysis of the average methylation ratio of NKX2.2 in CRC cell lines, treated with demethylation by 5-Aza-2'-deoxycytidine (5-AZC) (0 µmol/L vs 5 µmol/L vs 10 µmol/L). *p<0.05; **(C)** SYBR analysis of the relative mRNA level of APC2 in CRC cell lines treated with 5-AZC (0 µmol/L vs 5 µmol/L vs 10 µmol/L), *p<0.05; **(D)** Western blot analysis of the protein level of NKX2.2 in CRC cell lines treated with 5-AZC (0 µmol/L vs 5 µmol/L vs 10 µmol/L), p<0.05.

Then we investigated NKX2.2 methylation levels in CRC cell lines (HCT116, SW480, SW640, HT29, SW1116, LOVO) and reference cell line HEK-293T. It showed that the methylation levels of CRC cell lines were obviously higher than reference cell line HEK-293T (p < 0.05, Figure 4A). To confirm that the downregulation of transcription levels in cell lines was attributed to epigenetic hypermethylation, we treated cells with 5-AZC (0 μmol/L vs 5 μmol/L vs 10 μmol/L). The Sequenom MassArray results showed a gradual reduction in NKX2.2 methylation over 72 hours following treatment (p < 0.05, Figure 4B). Meanwhile, its mRNA and protein expression were found to gradually but significantly increase (p < 0.05, Figure 4C, 4D). These data suggests that NKX2.2 hypermethylation may be responsible for the downregulation of mRNA and protein expression in CRC.

### NKX2.2 suppresses cell proliferation in CRC cells

**Figure 5:**
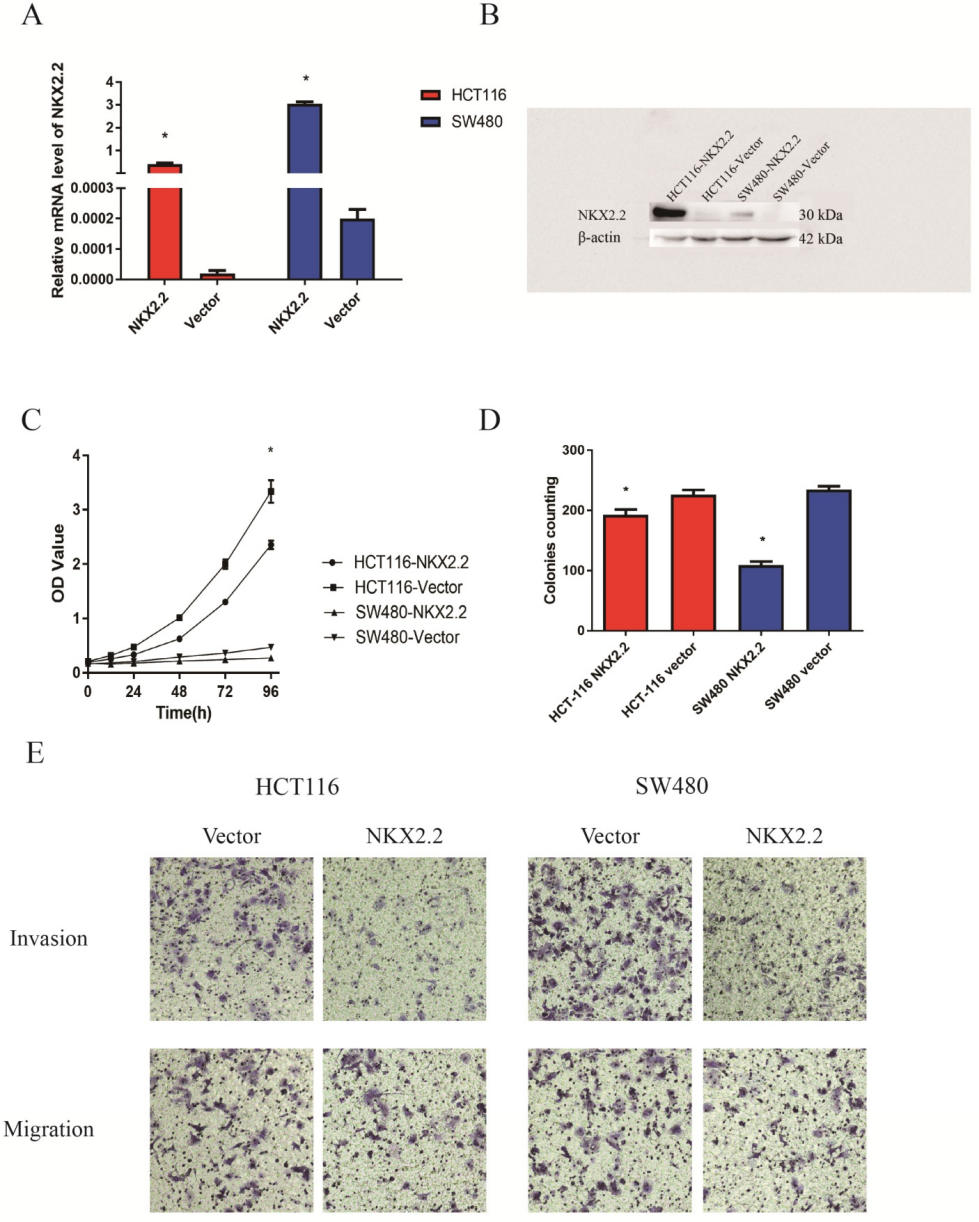
** (A and B)** Expression of NKX2.2 by RT-PCR (A) and western blot (B) analysis in Vector- and NKX2.2-transfected HCT116 and SW480 cells; **(C)** CCK8 assays showed that cell proliferation was significantly enhanced in vector-transfected HCT116 and SW480 cells than NKX2.2-transfected HCT116 and SW480 cells (*p<0.05); **(D)** Representative colony formation assay in vector- and NKX2.2-transfected HCT116 and SW480 cells (*p<0.05); **(E)** Cell invasion and migration assays revealed that the abilities of migration and invasion were weakened after transfected with NKX2.2 (p<0.05).

The expression of NKX2.2 by RT-PCR and western blot analysis in Vector- and NKX2.2-transfected HCT116 and SW480 cells have been showed in Figure 5A and 5B. To explore the effects of NKX2.2 on cell proliferation, cell viability was detected by CCK8 assays. Cell viability was significantly reduced after restoration of NKX2.2 expression in HCT116 and SW480 cells (both p < 0.05, Fig. 5C). Colony formation assay was performed to evaluate the effect of NKX2.2 on clonogenicity. The clone numbers were 192.66 ± 9.07 vs. 226.33 ± 13.50 in HCT116 group (pEZ-M35-NKX2.2 vs pEZ-M35-Vector) and 109.00 ± 10.53 vs. 234.66 ± 9.86 in SW480 group (pEZ-M35- NKX2.2 vs pEZ-M35-Vector) after overexpression of NKX2.2 (both p < 0.05, Fig. 5D).

### NKX2.2 inhibits CRC cell invasion and migration

To evaluate the effects of NKX2.2 on cell migration and invasion, the method of transwell assay was used. The number of migration and invasion cells decreased significantly after the re-expression of NKX2.2 in HCT116 group (pEZ-M35-NKX2.2 vs pEZ-M35-Vector) and SW480 cells group (pEZ-M35-NKX2.2 vs pEZ-M35-Vector) (p < 0.05, Fig. 5E).

## Discussion

DNA methylation was regarded to regulate the changes in expression of genes that make by adjusted interactions between DNA and messenger RNAs. In the past decades, methylation modification in CRC pathogenesis was demonstrated powerfully 2. It was reported that NKX2.2 was dysregulated in a variety of cancers. However, the possible molecular mechanism of NKX2.2 inactivation or epigenetic silencing in CRC remains unclear. Here we report that (1) NKX2.2 was hypermethylated in CRC tumor tissues and cell lines. An inverse correlation was observed between the NKX2.2 methylation and its transcriptional levels. (2) No significant association was found between the NKX2.2 methylation and sex, age, tumor differentiation, TNM stage, CEA, CA199, and fecal occult blood. (3) Furthermore, NKX2.2 hypermethylation showed a trend but not statistical significance for predicting poor overall survival in CRC patients. (4) NKX2.2 suppressed cell proliferation, colony formation, and inhibited tumor invasion and migration in CRC cells.

Previous studies have reported that NKX2.2 was methylated in carcinomas. Bhat et al. reported that NKX2.2 served as useful biomarkers for the early detection and clinical management of cervical cancer by analyses of next generation sequencing 16. Moreover, CpG islands in homeobox of NKX2-2 gene were significantly more methylated in Luminal A tumors than non-luminal subtypes 17. Our study found that NKX2.2 was hypermethylated in CRC tissues and 5-AZC restored its mRNA and protein expression in CRC cells, which was in consistent with previous findings, suggesting that NKX2.2 methylation might be common occurrence in the development of CRC.

NKX2.2 was a sensitive and specific diagnostic biomarker for the gastrointestine and pancreatic neuroendocrine tumors 13. It was demonstrated moderate or strong nuclear positivity in 91.2% of the Ewing sarcoma 18. NKX2.2 was a useful immunohistochemical marker for Ewing sarcoma, and that the combination of CD99 and NKX2.2 was a powerful diagnostic tool that can differentiate Ewing sarcoma from other small round cell tumors 19**.** NKX2.2 had high sensitivity (100%) and moderate specificity (85%) for the diagnosis of Ewing sarcoma in cytologic material staining, which was most commonly observed in small cell carcinoma (80%) and well differentiated neuroendocrine tumor (45%). Its staining was absent in melanoma, adenoid cystic carcinoma, and lymphoproliferative neoplasms 20. In consistent with previous studies, we demonstrated that NKX2.2 methylation showed good sensitivity and specificity in CRC, suggesting that it may be a good diagnostic biomarker for CRC patients. Besides, NKX2.2 hypermethylation showed a trend but not statistical significance for predicting poor overall survival in CRC, which may be associated with small sample size and incomplete follow up. A larger study with sufficient sample size would be valuable to verify the prognostic value of NKX2.2 methylation in the future.

The molecular mechanism of NKX2.2 in carcinomas was unclear. Some scholars found that NKX2-2 contributes to oncogenic transformation in Ewing's sarcoma, while downregulation of NKX2-2 correlates with increased tumor malignancy in glioblastoma 21-22. EWS/FLI and NKX2.2 repressed genes activated by ZEB2, which was shown to block Ewing sarcoma epithelialization 23. Sirt2 was a novel in vivo downstream target of NKX2.2 and enhanced oligodendroglial cell differentiation. Downregulation of the homeodomain transcription factor NKX2.2 was correlated with increased tumor malignancy 24. NKX2.2 overexpression by glioma-initiating cells induced oligodendroglial differentiation and suppressed self-renewal capacity. By contrast, NKX2.2 downregulation in mouse neural stem/progenitor cell accelerated glioblastoma formation. Importantly, the inhibitory effects of NXK2.2 on GIC self-renewal were conserved in human cells 22. NKX2.2 was a bona fide tumor suppressor for osteosarcoma. Moreover, overexpression of NKX2-2 decreased the migration, invasion, proliferation and colony formation of osteosarcoma cells in vitro and suppressed tumor growth and metastasis in vivo. NKX2.2 acted as a tumor suppressor partially by mediating the transcriptional downregulation of COL5A2, PLAU, SEMA7A and S1PR1 genes 15. NKX2.2 inhibited transcription of lymphoid NKL homeobox gene MSX1 and activates expression of basic helix-loop-helix factor NEUROD1 which may disturb B-cell differentiation processes via reported interaction with TCF3/E2A in B-cell malignancies 12. Elevated NKX2.2 expression in glioma initiating cells reduced their self-renewable ability and exhibited an antagonistic character to glioma proliferation. In vivo investigations in glioma initiating cells also supported the inhibitory effects of NKX2.2 in glioma malignancy 10. Our study was in line with previous studies, suggesting that the absence of NKX2.2 in CRC was attributed to methylation mechanism, leading to increase of cell proliferation, invasion and metastasis.

Some limitations of this study need to be acknowledged. Firstly, the number of CRC patients in the study was insufficient, and large cohort validation of NKX2.2 methylation needs to be completed. Moreover, the survival data is censored, which could be analyzed in the future. Finally, the detailed molecular carcinogenetic pathway mechanism was not elucidated, which deserves to be explored further.

## Conclusions

In conclusion, this present study indicates that NKX2.2 is hypermethylated and demonstrates a novel and important role of NKX2.2 as a tumor suppressor in CRC.

## Figures and Tables

**Table 1:** Relationship between NKX2.2 methylation and clinicopathological parameters in CRC

Clinical parameters	N (N1%)	The average methylation of NKX2.2	
		mean±SD	p
**Sex**			
Male	12(%)	0.21±0.07	0.11
Female	8 (%)	0.28±0.10	
**Age (years)**			
≥50	10 (%)	0.26±0.06	0.21
<50	10 (%)	0.22±0.11	
**Differentiation**			
Well	13(%)	0.24±0.10	0.81
Poor	7 (%)	0.23±0.08	
**TNM**			
I+II	12 (%)	0.25±0.10	0.63
III+IV	8 (%)	0.23±0.06	
**Tumor**			
3	11 (%)	0.23±0.09	0.61
4	9 (%)	0.25±0.09	
**Lymph node metastasis**			
Positive	7 (%)	0.23±0.07	0.77
Negative	13 (%)	0.24±0.10	
**Distant metastasis**			
Positive	4 (%)	0.23±0.14	0.8
Negative	16 (%)	0.24±0.10	
**CEA (ng/ml)**			
0-4.9	8 (%)	0.24±0.10	0.9
≥5	12 (%)	0.24±0.09	
**CA199**			
>37ku / L	5 (%)	0.24±0.11	0.97
≤37ku / L	15 (%)	0.23±0.09	
**Fecal occult blood**			
Positive	16 (%)	0.24±0.07	0.92
Negative	4 (%)	0.19±0.15	

CRC: colorectal cancer; CEA: carcinoembryonic antigen.
